# The Role of Vitamin D in Supporting Health in the COVID-19 Era

**DOI:** 10.3390/ijms23073621

**Published:** 2022-03-25

**Authors:** Alice Albergamo, Giulia Apprato, Francesca Silvagno

**Affiliations:** Department of Oncology, University of Torino, 10126 Torino, Italy; alice.albergamo@edu.unito.it (A.A.); giulia.apprato@unito.it (G.A.)

**Keywords:** vitamin D, COVID-19, inflammation, oxidative stress, redox imbalance, immunomodulation, vitamin D deficiency, vitamin D supplementation, mitochondrial uncoupling

## Abstract

The genomic activity of vitamin D is associated with metabolic effects, and the hormone has a strong impact on several physiological functions and, therefore, on health. Among its renowned functions, vitamin D is an immunomodulator and a molecule with an anti-inflammatory effect, and, recently, it has been much studied in relation to its response against viral infections, especially against COVID-19. This review aims to take stock of the correlation studies between vitamin D deficiency and increased risks of severe COVID-19 disease and, similarly, between vitamin D deficiency and acute respiratory distress syndrome. Based on this evidence, supplementation with vitamin D has been tested in clinical trials, and the results are discussed. Finally, this study includes a biochemical analysis on the effects of vitamin D in the body’s defense mechanisms against viral infection. In particular, the antioxidant and anti-inflammatory functions are considered in relation to energy metabolism, and the potential, beneficial effect of vitamin D in COVID-19 is described, with discussion of its influence on different biochemical pathways. The proposed, broader view of vitamin D activity could support a better-integrated approach in supplementation strategies against severe COVID-19, which could be valuable in a near future of living with an infection becoming endemic.

## 1. Introduction

### 1.1. Vitamin D Role on Human Health at the Time of COVID-19

Even though the vaccines against SARS-CoV-2 infection are available today, it seems likely that the evolution of the pandemic to endemic infection will create a forced coexistence between the virus and the world population. Strategies of protection and improvement of general health will make us more resistant to the serious consequences of the disease. Vitamin D exerts a wide range of physiological functions and plays a central role in human health due to its transcriptional activity over more than 1000 genes in most human tissues and cell types. Vitamin D deficiency, which is associated with levels of 25(OH)D3 below 20 ng/mL, is a global health problem, and it is generally estimated that more than three billion individuals in the world have vitamin D deficiency or insufficiency (between 21 and 29 ng/mL). Vitamin D status is reflected by the level of the circulating metabolite 25(OH)D3, and, according to the Endocrine Society, the optimal 25(OH)D3 plasma levels are in the range of 30–50 ng/mL [1]. The optimal values proposed by the guidelines do not necessarily guarantee the efficacy of the hormone due to the variable synthesis or catabolism of the active form of vitamin D in each person, and, therefore, the optimal levels compared to deficiency are yet to be established in relation to individual status. Indeed, in clinical trials, the optimal serum concentration of 25(OH)D3 is sometimes fixed at different values: between 30 and 60 ng/mL [2,3,4,5].

Moreover, not only are the levels of the hormone critical, but also the individual molecular response to vitamin D activity must be adequate; indeed, the general population can be divided into good and poor responders to the vitamin [1,6]. Several studies reported the impact of vitamin D deficiency on many diseases. This review aims at updating the link between vitamin D and COVID-19, which has been proposed in several recent studies. The role exerted by vitamin D in the inflammation and immune responses to respiratory viruses is described with a special focus on lung inflammation and cytokine release syndrome. Due to the central role exerted by inflammation in the severity of COVID-19 symptoms, this review discusses the evidence supporting or questioning the impact of vitamin D deficiency on COVID-19 outcomes and describes the results of vitamin D supplementation evaluated in COVID-19 patients. Finally, the biochemical analysis of the anti-inflammatory and antioxidant properties of vitamin D suggests future implementation strategies for increasing the efficacy in alleviating severe COVID-19 or easing long COVID syndrome in a future, persistent, endemic disease.

### 1.2. Vitamin D Synthesis and Activity

Vitamin D, also known as calcitriol or active vitamin D3 (1,25(OH)_2_D3), is a fat-soluble steroid that performs multiple functions aside from calcium and phosphate homeostasis regulation. Vitamin D can be obtained either from the diet or by UV-mediated two-step synthesis; in this latter case, it is produced in the skin from 7-dehydrocholesterol and subsequently converted into vitamin D3 or cholecalciferol. In order to become fully biologically active, cholecalciferol needs to be further hydroxylated in two positions, respectively, one and twenty-five; the first modification occurs in the liver, where vitamin D is hydroxylated by cytochrome P450 isoforms (mainly CYP2R1) into 25-hydroxyvitamin D3 (25(OH)D3 or calcidiol), and, afterward, in the kidney, where CYP27B1 performs the second hydroxylation, leading to the fully active 1,25-hydroxyvitamin D3 [7]. This latter enzyme, CYP27B1, is primarily found in the proximal renal tubule, but it is also expressed in several extra-renal sites, such as macrophages, dendritic cells (DCs), monocytes, B and T cells, and in epithelial cells, bone, and parathyroid glands [7]. The presence of vitamin D metabolic enzymes in non-classical tissues is crucial since the active hormone is characterized by quite a short half-life, and the calcitriol levels required to exert local activities in immune cells are higher than the physiological serum levels [8]. Cholecalciferol is the most commonly supplemented form of vitamin D, due to the activity of liver and tissues being able to convert the precursor into the active calcitriol form.

Like the other steroid hormones, calcitriol exerts its function through the binding of a nuclear receptor known as VDR (vitamin D receptor) that, upon binding, modulates the expression of its target genes. After the heterodimerization with the retinoid X receptor (RXR), VDR recognizes and binds specific DNA sequences known as vitamin D response elements (VDREs), switching on or off the transcription of the target genes [7] depending on the presence of additional coregulators and corepressors which are recruited in situ and are tissue-specific. Not only are the VDR target genes quite heterogeneous, but the receptor itself can be found expressed in many different tissues, reflecting the involvement of vitamin D in the regulation of multiple processes. Indeed, besides VDR expression in classical target tissues, such as bones, kidney, and intestine, where the hormone regulates bone mineralization, calcium, and phosphate absorption, respectively, VDR is found in non-classical target tissues too. In immune cells, for instance, it works as an immunomodulator, while, in the reproductive system, the endocrine system, muscles, the brain, skin, and liver, it exerts other functions [9]. Considering the wide heterogeneity of genes controlled by VDR, and its tissue-specific function, the crucial role of vitamin D in the maintenance of human health appears clear, and, therefore, the correlation between the hormone deficiency and the increased risk of developing certain cancers and several autoimmune and infectious diseases can be easily explained [10,11,12]. Through VDR binding, the vitamin exerts its control over a large set of genes, contributing to cellular differentiation [13], xenobiotic detoxification [14], immunity [15], and metabolism as well [16].

Moreover, besides the genomic regulation, the hormone exerts, through its receptor, non-genomic effects as well [17,18,19]. In the past years, since VDR mitochondrial localization was reported in the literature for the first time [20], several works tried to elucidate its role in the organelle. Our previous works [21,22], as well as others [23], demonstrated that, through VDR binding, vitamin D modulates mitochondrial respiration in cancer cells, keratinocytes, adipocytes, immune cells, and other cells.

## 2. Vitamin D as Immunomodulator and Immunostimulant against Respiratory Viruses

In addition to skeletal homeostasis, calcitriol acts as an immunomodulator in both innate and adaptive immune responses, exerting a specific function according to the cell type where the hormone is produced and acts. Indeed, both VDR and CYP27B1 are expressed in almost all immune cells, and their expression is modulated by the presence of an infection. In monocytes and macrophages, which represent the first line of defense against microbial pathogens, CYP27B1 is strongly induced by interferon-gamma (IFN-γ), an important pro-inflammatory cytokine produced in the case of microbial infections, and both CYP27B1 and VDR are induced by the toll-like receptors (TLRs) signaling pathway, which is activated in the case of viral or tuberculosis infections [24,25]. The local production of calcitriol in these innate immune cells increases the production of chemokines, fundamental recruiters of innate and adaptive immune cells, and antimicrobial proteins, such as cathelicidin LL-37 [1,26,27,28]. In particular, cathelicidin exerts antibacterial and antiviral activity against respiratory viruses, disrupting, respectively, the microbial membrane and the viral envelope [1,26,29]. Active vitamin D also promotes the phagocytic activity of macrophages and DCs, which consists of the endocytosis of the pathogen and further digestion of the pathogenic antigens. Moreover, it reduces the production of pro-inflammatory cytokines, such as tumor necrosis factor-α (TNF-α), INF-γ, interleukin (IL)-6, and IL-1, while increasing anti-inflammatory cytokines such as IL-10 [30,31,32]. In particular, a recent study focused on the synergic effect of calcitriol and beta-glucans, another well-known immunomodulator, on macrophage phagocytic activity and cytokines production; the authors concluded that both molecules converge on the same pathway and that the enhanced production of immunomodulatory cytokines observed after cotreatment with the two molecules was mediated by the increased vesicular acidification [33].

VDR expression, as well, seems to be controlled by immune signals, increasing or decreasing according to the cell type, highlighting the specific dependence of immune cells on calcitriol presence and activity. In fact, both CYP27B1 and VDR are induced upon stimulation of several immune cell types, namely macrophages [8,34,35,36,37,38], T cells [35,37], and B cells [36], whereas, in DCs, CYP27B1 expression and activity increase as DCs differentiate towards a mature phenotype, and VDR decreases in mature DCs compared to immature DCs or monocytes; the reciprocal organization of CYP27B1 enzymes and VDR during cell maturation can limit over-stimulation of T cells [35,36]. In particular, in antigen-presenting cells, such as macrophages and DCs, VDR seems to potentiate the initiation of the immune response, acting on the phagocytosis and pathogen digestion steps while inhibiting T cells activation through the repression of the genes involved in the antigen processing and presentation mechanisms [8]. Moreover, calcitriol seems able to foster the production of reactive oxygen species (ROS) and inducible nitric oxide synthase (iNOS): crucial species involved in killing pathogens in both monocytes and macrophages [39]. These effects are thought to be achieved through the modulation of class III phosphatidylinositol 3-kinase complex (PI3KC3) [39].

During the DCs’ maturation process, the production of costimulatory molecules and the expression of the major histocompatibility complex (MHC) class II on the cell surface are inhibited by active vitamin D through autocrine and paracrine signals; B and T cells activation is altered as well [40,41,42]. Indeed, locally produced calcitriol seems to induce a tolerogenic phenotype of adaptive immune cells. In particular, it suppresses T cells proliferation and alters the activation of T helper (Th), inhibiting the differentiation of Th1 and Th17 phenotypes and promoting Th2 cells [8,43,44,45]. The suppression of the pro-inflammatory state is achieved by the hormone promoting the differentiation of regulatory T cells (Treg), which are an immunosuppressive population downregulating the induction and proliferation of other T cells [46]. According to several works, this tolerogenic environment could be one of the reasons why vitamin D helps to protect against autoimmune diseases [1]. In cytotoxic T lymphocytes (CTL) as well, VDR is upregulated in case of infection, and CYP27B1 is always expressed; however, the effects of the vitamin on the proliferation, differentiation, and functions of these cells remain unelucidated [47,48]. Inactive B cells lack VDR, and, once activated, they upregulate the receptor to proliferate in the same manner as T cells; B cells express CYP27B1 as well, allowing the local production of the hormone that seems to be crucial for the regulation of their activity [49]. Indeed, it is suggested that calcitriol negatively regulates B cell activity and differentiation in plasma cells, reducing autoantibody production too and, hence, protecting from autoimmune disorders [44,49].

A recent study identified a shortlist of the major targets of active vitamin D in stimulated monocytes and lymphocytes. Among them, there are some antimicrobial peptides, several transcriptional factors, transmembrane proteins as TLRs, cytoplasmic proteins, such as mitogen-activated protein kinase (MAPK), and secreted proteins [15]. Among the genes that appear to be strongly induced by calcitriol, cathelicidins are involved in the acute response to pathogens and are thought to mediate the vitamin-D-enhanced innate immunity to respiratory infections. Indeed, the promoter of human cationic antimicrobial peptide of 18 kDa (hCAP-18), the sole form of cathelicidin found in humans, seems to contain a VDR-responsive element (VDRE), suggesting that the hCAP-18-enhanced production, in the case of microbial infection, depends on vitamin D-VDR activation [50,51]. β-defensin 2, another crucial antimicrobial peptide able to induce immune cells chemotaxis seems to be upregulated by vitamin D-VDR as well. Indeed, the DEFB4 (defensin beta 4A) gene proximal promoter region contains a VDRE [50].

Several studies focused on calcitriol effects on host immune responses to respiratory viruses. Respiratory viruses, through the recognition and activation of TLRs, trigger cytokine production which, in turn, results in the enhanced production of 1,25(OH)_2_D3 [52]. Through this mechanism, active vitamin D is thought to be able to modulate the immune response to better respond to these pathogens. Calcitriol treatment of primary human bronchial epithelial cells infected with rhinovirus 16 (RV-16) increased the secretion of pro-inflammatory cytokines, including C-X-C motif chemokine ligand 8 (CXCL8) and CXCL10, crucial in the recruitment of macrophages, neutrophils, and T cells in the site of infection [53]. Active vitamin D seems to inhibit respiratory syncytial virus (RSV) infection as well. A vitamin D pre-treatment of primary human tracheobronchial epithelial cells infected with RSV resulted in increased NF-kB inhibitor IκBα, which prevented NF-kB translocation into the nucleus and subsequent binding to DNA. Although NF-kB inhibition limited the production of antiviral molecules, such as interferon-beta (IFN-β) and CXCL10, resulting in a reduced inflammatory response, the viral replication and load remained constant, suggesting a potential role of the active vitamin in reducing RSV immunopathology [54]. Moreover, calcitriol induced the secretion of both antiviral peptides, cathelicidin and β-defensin 2, reducing RSV viral cellular entry and, therefore, the spread of infection [55].

Calcitriol pretreatment was also found to be effective in preventing immunopathology in alveolar cells infected with the influenza virus. Indeed, the vitamin was demonstrated to decrease the production of pro-inflammatory factors, such as tumor necrosis factor-alpha (TNF-α), IFN-β, CXCL8, and interleukin (IL)-6 [56], decreasing the risk of severe complications, such as the cytokine storm associated with a hyperinflammatory response [57].

## 3. Vitamin D Anti-Inflammatory Properties: A Role in Inflammatory Pulmonary Diseases

Since active vitamin D is a crucial immunomodulator and contributes to the creation of a tolerogenic environment, it decreases the risk of developing inflammatory and autoimmune diseases [58,59]. Both in vitro and in vivo studies confirmed vitamin D anti-inflammatory properties; in particular, VDR absence was associated with increased activity of NF-kB, a key transcription factor playing a crucial role in several inflammatory diseases and chronic inflammatory states [60]. Calcitriol promoted human bronchial cell survival following LPS-induced inflammation by downregulating NF-kB, TNF-α, IL-1ß, IL-6, and IL-12, indicating that active vitamin D has the potential to manage lung inflammation [61]. A systematic review confirmed the anti-inflammatory effects of the hormone in both human cell lines and PBMCs [62], and in vivo studies demonstrated that vitamin D administration decreased the development of type 1 diabetes, inflammatory arthritis, autoimmune encephalomyelitis, and thyroiditis [63]. The activity of calcitriol could be crucial in moderating the inflammatory pathologies concurring with severe COVID-19 outcomes; thus, as part of a better general protection, active vitamin D could decrease the severity of the disease at times of increased risk of exposure to the virus.

Vitamin D deficiency in humans is associated with pathologies characterized by an inflammatory background. The link is particularly evident in lung inflammation, which is prodromal to many diseases when inflammation becomes chronic; exacerbated lung inflammation is often involved in the worsening of the COVID-19 disease. Active vitamin D was demonstrated to possess inhibitory effects on pulmonary inflammation by strongly influencing the functions of inflammatory cells, including DCs, monocyte/macrophages, T cells, and B cells, and the integrity of structural epithelial cells [64]. Vitamin D deficiency is related to several lung pathologies, including respiratory distress syndrome, alveolar inflammation, and epithelial damage. A link between vitamin D status and respiratory morbidity was revealed by several studies on children [65,66,67], adults, and the elderly [68,69]. It was clearly demonstrated that low vitamin D status was associated with inflammation in patients with chronic obstructive pulmonary disease (COPD) [70].

The signs of lung inflammation induced in mice by vitamin D deficiency were diminished by subsequent supplementation with the hormone in terms of macrophage and neutrophil numbers [71]. Moreover, total IgG levels in the bronchoalveolar lavage fluid were greater in mice fed the vitamin-D-containing diet, which may be explained by increased activation of B cells in the airway-draining lymph nodes. These findings highlight the importance of vitamin D supplementation aimed at reducing the severity of asthma and other chronic lung diseases, as demonstrated by a recent meta-analysis. The work, indeed, suggests that vitamin D supplementation may reduce the likelihood of asthma exacerbation [72,73].

Moreover, epidemiological studies identified a relation between hypovitaminosis D, asthma insurgence, and severe exacerbation [74]. In fact, asthma is a long-term inflammatory disease characterized by airflow obstruction and bronchial hyperresponsiveness caused by an inappropriate response and activation of Th2 [75,76]. Although the mechanisms of action of vitamin D in the context of the pathology are still not clear, the hormone is thought to act in the airways by increasing the production of IL-10 and by limiting the production and secretion of the pro-inflammatory cytokines produced by Th2 [77,78]. COPD is another pathological condition characterized by the narrowing of the airways. Chronic bronchitis and alveoli emphysema are the consequences of the constant and chronic inflammation of the airways which are populated by neutrophils and macrophages that produce and release pro-inflammatory cytokines. The deterioration of lung function is mainly due to the unbalanced activity of matrix metalloproteinases (MMPs), especially MMP-9, which is stimulated by TNF-α produced by alveolar macrophages. In this context, active vitamin D increases the production of IL-10 that, in turn, increases the activity of the tissue inhibitor of metalloproteinase (TIMP)-1 and inhibits TNF-α secretion, decreasing MMP-9 activity and production, restoring the lost equilibrium [79,80]. Moreover, calcitriol is indirectly capable of protecting from both COPD and the asthma exacerbation often triggered by viral or bacterial infections; indeed, vitamin D induction of cathelicidin expression helps to decrease the pathogen load and, hence, reduce the frequency of worsening due to respiratory tract infections [76].

## 4. Vitamin D and Respiratory Tract Infections

Considering the crucial role of vitamin D in the modulation of immune responses, a correlation between vitamin D deficiency and susceptibility to respiratory infections was identified, in particular regarding *Mycobacterium tuberculosis*, Gram-negative bacteria, and viral infections [52,78].

### 4.1. Bacterial Infections

A meta-analysis reported that low levels of serum vitamin D were associated with increased susceptibility to tuberculosis; moreover, the administration of vitamin D to tuberculosis patients was demonstrated to greatly improve the overall conditions of the patients compared to the placebo group [76]. In children, some studies indicated a significant association between tuberculosis and vitamin D deficiency, and the results indicated that vitamin D supplementation may be beneficial for tuberculosis treatment and prevention, although studies in children are limited with small sample sizes [81]. The increased response of the tuberculosis patients following vitamin D administration can be explained since the hormone induces cathelicidin LL-37 production in macrophages and in bronchial epithelial cells, which promotes the killing of the pathogen [76]. Moreover, ROS mediate the effect of active vitamin D, because their neutralization prevents the antimycobacterial activity of calcitriol-treated macrophages [82]. Active vitamin D demonstrates a broad range of antimicrobial actions, including the suppression of hepcidin and the consequent reduction of intracellular iron necessary for bacterial growth and the stimulation of neutrophils [83]. The anti-inflammatory effects of vitamin D during bacterial infection were demonstrated, such as the decreased cytokine production in ex vivo PBMC and monocytes treated with bacterial ligands and calcitriol, as was the protection against bacterial infection due to the increase of CD14 expression [84].

Like immune cells, several respiratory epithelial cells were demonstrated to express CYP27B1, allowing the local synthesis of the hormone, highlighting the importance of vitamin D in the respiratory tract, especially in the defense against respiratory pathogens [54]. In this regard, active vitamin D was demonstrated to be crucial in the maintenance of intact physical barriers, the first line of defense against the pathogens, reinforcing the tight, adherens and gap junctions via the induction of E-cadherin [85].

### 4.2. Viral Infections

Several studies showed a link between vitamin D deficiency and an increased risk of respiratory tract infections [78]. Therefore, this hormone is thought to play a key role in reducing the risk of viral infections starting from influenza viruses. Grant et al. analyzed the mechanisms through which active vitamin D reduces the risk of the common cold. At first, this hormone helps to keep the various cellular junctions intact against virus attack. It also stimulates innate immunity through the induction of antimicrobial peptides, such as defensins and cathelicidins, and inhibits the cytokine cascade, reducing the production of pro-inflammatory Th1 cytokines such as INF-γ and TNF-α. Finally, active vitamin D modulates the adaptive immune response by inhibiting the activity of Th1 cells and promoting the production of cytokines by Th2 cells, which suppresses inflammation [86].

Viral acute respiratory infections (ARI) are a global major health problem characterized by high morbidity and mortality due to the limited availability of effective antiviral drugs and vaccines [52]. Several clinical trials were carried out to understand whether supplementation of vitamin D can prevent ARI; although the results were quite heterogeneous, a successive individual participant data meta-analysis of randomized, controlled trials found that vitamin D supplementation (cholecalciferol or ergocalciferol) reduced the risk of experiencing at least one acute respiratory tract infection [87]. Many studies suggested an association between vitamin D insufficiency during the winter period, caused by reduced sun exposure, and the seasonal incidence variations in influenza and pneumococcal, community-acquired pneumonia [74]. In fact, ecological studies suggested increasing the 1,25(OH)_2_D3 concentration through cholecalciferol supplementation in winter with the aim of reducing the development of influenza [86]. A similar association was confirmed by several placebo-controlled, double-blinded studies; in particular, Laaksi et al., in a randomized, controlled trial, found that a group of voluntary, young Finnish men, supplemented with cholecalciferol for the period of observation (6 months), reported fewer ARIs compared to the placebo control group [88]. Camargo and colleagues, a few years later, observed that children with vitamin D deficiency, not treated with vitamin supplementation, were characterized by a greater susceptibility to acute respiratory infections [89]. VDR-knockout mice further confirmed the protective and crucial role of vitamin D in fighting acute infections [74].

## 5. Vitamin D and COVID-19

### 5.1. The Cytokine Release Syndrome

Among the respiratory viruses, SARS-CoV-2 is the latest coronavirus discovered and the most studied in recent years since it causes the pathology known as COVID-19. The clinical symptoms of the patients affected by COVID-19 range from mild respiratory diseases, including fever, cough, and dyspnea, to severe acute respiratory diseases, which can culminate in severe pneumonia and in acute respiratory distress syndrome (ARDS), a severe, life-threatening condition. The severity of the disease mainly depends on the presence of the so-called “cytokine storm”, an exacerbated, inflammatory cytokine release due to a potentially fatal, excessive immune response [90]. After binding SARS-CoV-2 spike proteins to the cellular ACE2 (angiotensin-converting enzyme 2) and entering respiratory epithelial cells, the pathogen causes a Th1 excessive release of cytokines, including IL-6 and granulocyte-macrophage colony-stimulating factor (GM-CSF), which recruit and activate CD14^+^CD16^+^monocytes, which, in turn, produce IL-6, TNF-α, and other cytokines [91] and further recruit macrophages and neutrophils into the lung tissue. Neutrophil extracellular traps and the weak induction of interferon-γ (IFN-γ) further amplify the cytokines’ production and release [91,92].

It was reported that SARS-CoV-2 interferes with the normal balance of the main mediators of the RAS (renin–angiotensin system) pathway ACE/ACE2. The ACE enzyme converts angiotensin I (AngI) to angiotensin II (AngII), a vasoconstrictor peptide that binds to the AT1 receptor (angiotensin II receptor type 1). ACE2 acts as a counter-regulator because it reduces the levels of AngII by transforming it into another molecule called Ang(1-7), which binds to the Mas receptor [93,94,95,96,97,98]. Therefore, there are two pathways in the renin–angiotensin system (RAS): the ACE–AngII–AT1R axis, which causes vasoconstriction, proliferation, inflammation, and apoptosis, and the non-canonical ACE2–Ang(1-7)–MasR pathway, which neutralizes the different functions of AngII, leading to vasodilation and the exertion of anti-inflammatory and anti-apoptotic effects [98]. SARS-CoV-2 induces the Ang II/AT1R pathway, resulting in severe COVID-19 consequences [99]. The downregulation of ACE2 and the imbalance between the renin–angiotensin system and ACE2–Ang(1-7)–MasR following COVID-19 infection was suggested to be participating in the pathogenesis of multiple-organ damage in this disorder [100].

Based on the anti-inflammatory properties of vitamin D, several studies assessed the levels of the hormone in COVID-19 patients and investigated the results of supplementation. It is important to distinguish between vitamin D levels as a risk factor for SARS-CoV-2 infection and vitamin D status as a promoter of health in COVID-19 patients; therefore, in this review, we separate and describe the studies investigating either the correlation with the severity of the disease or the correlation with infection risk.

### 5.2. Vitamin D Deficiency and COVID-19: Evidence of a Positive Correlation

Numerous studies showed a relationship between vitamin D status and unfavorable clinical courses from SARS-CoV-2. Recently, the scientific community hypothesized that COVID-19 may follow a seasonal pattern, like other respiratory viruses. Some studies showed a possible association between the development of SARS-CoV-2 infection and latitude [101,102,103], suggesting a possible protective effect of exposure to ultraviolet (UV) radiation. Solar UVB light is highly efficient in inactivating SARS-CoV-2 in aerosol form and on surfaces [104] and may modulate the diffusion of the SARS-CoV-2 epidemics, explaining the geographical and seasonal differences observed for this disease. In addition, higher ambient UVA exposure is associated with lower COVID-19-specific mortality [105], possibly through a nitric-oxide-mediated pathway, which could impact on virus replication and could mitigate endothelial damage. Moreover, at latitudes below 35 degrees on either side of the equator, it is estimated that the overall UVB radiation exposure is sufficient for the annual vitamin D synthesis necessary to avoid hypovitaminosis D. Vitamin D deficiency is common in the UK and southern European countries, whereas northern countries maintain optimal levels due to the widespread use of supplements and foods rich in calcitriol [106]. Since the diet is a source of vitamin D as well as UV radiation exposure, it gets difficult to interpret the variations of calcitriol levels in countries at different latitudes. Moreover, when assessing the possible link between deficiency of vitamin D and COVID-19 outcome, the heterogeneity that characterizes vitamin D individual response makes the evaluation uncertain. In patients with SARS-CoV-2, disease severity is strongly associated with the cytokine storm [91], and, considering the anti-inflammatory function of vitamin D, it is hypothesized that hypovitaminosis D may be involved in the development of severe complications of COVID-19, including hospitalization and intensive care. Several studies highlight the immunological effects of vitamin D in the pulmonary inflammatory process that characterizes the infection [107,108]. An observational study, which included COVID-19 intensive care unit (ICU) patients and non-ICU, hospitalized patients, showed that vitamin D deficiency (25(OH)D_3_ below 20 ng/mL) was associated with reduced numbers of NK cells (<100 cells/mL) and concluded that mild NK lymphopenia in patients suffering from vitamin D deficiency may be crucial in early, viral infections since NK cells are the first cellular barrier encountered by the pathogens [108]. Kloc et al. pointed out that the hyperinflammatory response triggered by pulmonary macrophages and myeloid-derived suppressor cells (MDSCs) causes ARDS, and these cells could be targets for vitamin D intervention because they express both VDR and metabolic enzymes for active vitamin D synthesis [107]. This evidence supports the effort of investigating whether severe hypovitaminosis D is a marker of poor COVID-19 prognosis and whether this condition is associated with higher lethality. The most significant studies analyzing the correlation between vitamin D levels and disease course are listed in Supplementary Table S1.

The inverse correlation between blood 25(OH)D3 concentrations and probability of hospitalization and mortality was demonstrated [109]; indeed, a plasma 25(OH)D3 level below 20 ng/mL nearly doubled the risk of hospitalization for COVID-19 infection [110], and pre-infection deficiency of vitamin D was associated with increased disease severity and mortality [111]. Another observational study of COVID-19 patients showed that patients with vitamin D deficiency had a higher rate of admission to intensive care, regardless of age and sex [112]. In this context, it is necessary to mention that severe vitamin D deficiency is not necessarily directly related to SARS-CoV-2 mortality, since hypovitaminosis alone may increase mortality risk in hospitalized patients. An Italian retrospective study reported that infected patients admitted to the respiratory intermediate care unit (RICU) with vitamin D levels <10 ng/mL had a 50% of probability of death, whereas, in patients with vitamin D ≥ 10 ng/mL, the mortality rate was reduced to 5% [113]. Another prospective, observational study of SARS-CoV-2-positive patients treated in a multidisciplinary ICU demonstrated that low 25(OH)D3 levels are associated with an increased risk of mortality; this was supported by the fact that patients with 25(OH)D3 < 15.2 ng/mL died within 20 ± 7 days from ICU admission, whereas patients with 25(OH)D3 levels ≥15.2 ng/mL died within 44 ± 7 days [114]. The increased mortality in patients with serum 25(OH)D3 < 10 ng/mL is consistent with that reported in other studies [115,116].

An extensive, systematic review and meta-analysis of multiple observational studies suggested that vitamin D deficiency/insufficiency increases susceptibility to severe COVID-19 [117]. This result was confirmed by other meta-analyses, which reported lower serum levels of vitamin D in patients with a poor prognosis compared with those with a good prognosis [118] and showed that vitamin D deficiency was associated with severity and higher mortality rate [119]. Similar results were obtained in the investigation of the inflammatory response of COVID-19 patients, which reported an inverse correlation between several inflammatory markers and 25OHD levels and confirmed that vitamin D levels were lower in the group with more severe disease and in non-survivor patients [120].

There are several factors that affect the status of vitamin D, including diet, skin pigmentation, the amount of time spent outdoors, and age. In particular, it was observed that 25(OH)D3 serum concentrations tend to decrease with age, showing a negative correlation with the increased mortality rate for COVID-19. Vitamin D deficiency in the elderly depends on several factors; in part, the deficiency can be caused by a decreased ability to synthesize cholecalciferol in response to UV-radiation exposure [121], but, mainly, it is due to the use of anti-hypertensive, anti-neoplastic, and anti-epileptic drugs. Indeed, the vast majority of these drugs activate the pregnane X receptor (PXR) or the constitutive androstane receptor (CAR), which, by increasing the expression of 24-hydroxylases, causes a reduction of the levels of 1,25(OH)_2_D3 [122,123,124]. A recent study conducted in a cohort of 75-year-old patients highlighted the correlation between low serum vitamin D levels and low PaO_2_/FiO_2_ values (arterial pO_2_/inspired oxygen fraction), an independent risk factor for death in elderly COVID-19 patients which is used to assess the severity of hypoxemia [125].

In addition to aging, all the pre-existing conditions characterized by vitamin D deficiency, including obesity, diabetes mellitus, hypertension, cardiovascular disease, chronic kidney diseases, and stroke, were found to be significant predictors for severity of COVID-19 disease independent of age, sex, and body mass index (BMI). The study found significantly lower 25(OH)D levels among SARS-CoV-2-positive patients as compared to negative, hospitalized patients. Although severe vitamin D deficiency only proved to be a significant predictor of death in the unadjusted model, the authors found an elevated risk even in the adjusted models [126].

Although these results all relate to an adult age group, low levels of vitamin D were even observed in younger COVID-19 patients with fewer comorbidities; hence, it can be assumed that vitamin D deficiency may be a crucial risk factor at any age, although vitamin D deficiency and SARS-CoV-2 spreading are less common in summer. In particular, Bayramoğlu et al. evaluated the relationship between serum vitamin D levels and the clinical severity of COVID-19 symptoms in a cohort of pediatric cases (1–18 years). It was shown that 25(OH)D3 positively correlated with lymphocyte count and negatively correlated with age, CRP, and fibrinogen levels; the authors concluded that vitamin D deficiency is associated with a six-fold increase in the moderate-to-severe clinical course in the pediatric cohort [127]. Overall, the results of these studies support the hypothesis that vitamin D deficiency, in any age group, is a potential risk factor in the occurrence of severe COVID-19 symptomatology.

Some studies show that the likelihood of contracting SARS-CoV-2 infection seems to be related to low levels of 25(OH)D3, although this correlation was not always found. The most significant studies investigating the correlation between vitamin D levels and risk of infection are listed in Supplementary Table S2. An Israeli, population-based study showed that the mean 25(OH)D3 serum levels were significantly lower in COVID-19-positive patients than in the negative ones [110]. Vitamin D deficiency/insufficiency increases susceptibility to COVID-19, as reported by two systematic reviews and meta-analyses [117,119]. Moreover, SARS-CoV-2 positivity is strongly and inversely associated with circulating 25(OH)D3 levels [128].

### 5.3. Vitamin D Deficiency and COVID-19: Lack of Correlating Evidence

Despite the previously cited publications, the correlation between vitamin D serum levels and the increased risk of severe COVID-19 is not always evident; in fact, some studies reported conflicting data. The analysis of the UK Biobank, including 449 subjects with SARS-CoV-2 infection, did not find a potential link between plasma 25(OH)D3 levels and the likelihood of infection, suggesting that measurement of 25(OH)D3 is not useful for assessing the increased risk of COVID-19 in clinical practice [129]. However, this study was characterized by several limitations, especially the long time between 25(OH)D3 measurement and COVID-19 testing (more than ten years); several studies employing the same database showed the same flaw [103,130,131].

Another study conducted on a cohort of Brazilian patients showed no significant difference in mean 25(OH)D3 level between men and women or between adults and the elderly (over 60 years) and no difference in vitamin D status between infected Brazilians with SARS-CoV-2 and uninfected controls [132]. As for ethnic differences in COVID-19, these studies suggest that clinical, environmental, socioeconomic, and cultural factors have greater relevance than vitamin D status in determining susceptibility to SARS-CoV-2 infections.

A literature data analysis showed that, in European countries, there is not a significant association between vitamin D deficiency, COVID-19 cases, and recoveries; moreover, the authors concluded that the prevalence of severe calcitriol deficiency is not associated with the COVID-19 epidemic [133]. However, the authors pointed out that there was a relationship between the COVID-19 mortality risk and both mild (<50 ng/mL) and severe deficiency (<30 ng/mL) of 25(OH)D3, as confirmed by another study [126]. Consequently, the same authors suggested that governments should implement preventive measures to raise awareness among the population of the risk of vitamin D deficiency even before solid data supporting its protective role in the pandemic were available [133].

Finally, only one study so far investigated the correlation between vitamin D levels and long COVID symptoms, reporting a lack of correlation with fatigue and reduced exercise tolerance [134]. However, the study was limited by the small number of patients with low levels of vitamin D and by the fact that the investigation was performed at a median time from initial infection of 79 days, without estimation of vitamin D levels variation from infection.

### 5.4. Concluding Remarks on Vitamin D Deficiency and COVID-19

From the analysis of the correlations reported so far, the discrepancy of conclusions leaves some doubts about the correlation between levels of vitamin D and risk of infection, whereas the role of vitamin D in protecting from severe disease and death is supported by the correlation studies demonstrating that patients with very low levels of vitamin D are more susceptible to a worse prognosis. Drawing general conclusions from the plethora of reported studies is made difficult by the heterogeneity of clinical protocols, different parameters analyzed, and several mentioned limitations; a glance at the provided Supplementary Tables S1–S3 actually clearly shows the heterogeneity of the discussed studies.

In our opinion, the reasons that undermine a general acknowledgement of the correlation between vitamin D levels and COVID-19 can be found in (1) the failure to distinguish between the role of vitamin D as a risk factor for infection and a risk factor for severe disease and (2) the concept of individual response to vitamin D [1,6] wherein it is difficult to establish in each individual which serum level of the hormone is sufficient to guarantee proper anti-inflammatory activity and protection from a viral infection, making the classification of patients and the interpretation of results complex. Larger studies and possibly the assessment of individual sensitivity are required to achieve a final consensus on this scientific issue and to support the encouraging positive correlations found. In addition, more correlation studies on long COVID are required, with a focused assessment of several persistent symptoms in a larger cohort with insufficient or deficient levels of vitamin D.

### 5.5. Vitamin D Deficiency in Acute Respiratory Distress Syndrome (ARDS)

In the most severe manifestations, SARS-CoV-2 infection may trigger bilateral interstitial pneumonia, which represents the first phase of severe lung inflammation and may evolve into the dangerous ARDS [61,135,136]. In particular, the inflammation affects the interstitium between the epithelial alveolar cells and the capillary membrane where the gas exchange takes place; after inflammatory cell infiltration, the alveoli functionality is compromised and, if the inflammation becomes chronic, the fibrotic phase takes over with the deposition of scar tissue that replaces the alveoli tissues. As a result of this condition, normal lung function is lost, and patients must be assisted with mechanical pulmonary ventilation. Moreover, ARDS may result in systemic inflammation followed by multi-organ, systemic failure [91].

Low levels of vitamin D are associated with numerous pulmonary diseases, including acute lung injury (ALI) and ARDS [61,137,138], and this may support the correlation between hypovitaminosis D and the severity of symptoms of COVID-19. An in vitro study conducted in lung epithelial cells demonstrated that 1,25(OH)_2_D3 inhibited LPS-induced inflammation by increasing cell viability and downregulating pro-inflammatory cytokines, including TNF-α, IL-1b, and IL-6 [61]. The study of Shi et al. showed that a lack of VDR signaling may reduce the pulmonary epithelial barrier defense through a decrease in occludin and ZO-1 expression, proteins that help to maintain the epithelial barrier cohesion, and may exacerbate LPS-induced lung injury. Indeed, in mice models, pretreatment with the vitamin D analog paricalcitol inhibited the build-up of chemokines and alleviated ALI by maintaining occludin and ZO-1 expression [137]. In addition to the anti-inflammatory activity of vitamin D, which helps to reduce the effects of cytokine storm [139], the possible trophic and anti-apoptotic role of physiological 1,25(OH)_2_D3 levels (100 nmol/L) emerged in alveolar cells, the main targets of ARDS. The same study of Dancer et al. showed that, in vitamin-D-deficient patients with ARDS, severe deficiency of 25(OH)D3 (<20 nmol/L) was associated with increased accumulation of extravascular water and increased permeability of the alveolar–capillary barrier, concluding that vitamin D might have protective effects at the pulmonary level [138]. These results suggest the possible association between hypovitaminosis D and the severe pulmonary complication of COVID-19 and how vitamin D can reduce this through its anti-inflammatory and anti-apoptotic functions.

Vitamin D status was also associated with the duration of mechanical ventilation and mortality in patients admitted with ARDS. Data analysis of a randomized, controlled trial from the ALVEOLI study revealed that severe vitamin D deficiency was common in patients with ARDS [140]. In fact, patients with 25(OH)D3 < 10 ng/mL were likely to be ventilated for nine days longer than patients with levels >10 ng/mL and showed a higher risk of 90-day mortality [140].

Since these results seemed to show an association between vitamin D deficiency and a worsening in COVID-19 ARDS patients, several studies tried to understand if calcitriol could at least ameliorate patients’ conditions. To investigate this association, it was necessary to monitor vitamin D status over the patients’ clinical course. In the study of Notz et al., 85% of patients with life-threatening COVID-19 ARDS had the 25-hydroxyvitamin D deficiency associated with low circulating levels of plasmablasts involved in the setting up of immune memory and in the build-up of antibodies. However, low 25-hydroxyvitamin D levels did not correlate with the clinical course changes. On the other hand, 1,25-dihydroxy vitamin D levels ≥20 pg/mL after 10 to 15 days of intensive care were associated with better PaO_2_/FiO_2_ ratios and significantly fewer days of mechanical ventilation, suggesting that optimal levels of vitamin D might be associated with a less severe COVID-19 prognosis [136].

These findings support the potentiality of vitamin D to reduce COVID-19 ARDS patients’ inflammation; however, it is crucial to keep in mind that vitamin D alone does not seem to protect against infection, but, through its anti-inflammatory and anti-apoptotic role, can reduce dangerous, life-threatening lung lesions [138].

## 6. Vitamin D Supplementation and COVID-19

### 6.1. Clinical Evidence

Considering that many observational studies demonstrated a likely association between low 1,25(OH)_2_D3 levels and a higher risk of severe COVID-19, vitamin D supplementation was proposed as a possible preventive or therapeutic approach. The most significant studies investigating the effects of supplementation are listed in Supplementary Table S3. These studies adopted quite heterogeneous protocols of treatment in terms of dosage, duration, and vitamin D form. Nevertheless, some general conclusions can be drawn.

Several studies used high doses of the precursor molecule cholecalciferol. The study of Rastogi et al. examined SARS-CoV-2-positive individuals who were mildly symptomatic or asymptomatic, with or without any comorbidities, who were administered with a short-term high dosage of cholecalciferol (60,000 IU daily) to reach 25(OH)D3 serum levels higher than 50 ng/mL. The results of this study showed that a greater proportion of vitamin-D-supplemented patients achieved SARS-CoV-2 RNA negativity before day 21 compared to vitamin-D-deficient individuals of the control cohort [141]. Levels of fibrinogen, a plasma glycoprotein that increases during infections and inflammation, were also measured, and a significant reduction was observed in patients achieving 25(OH)D3 > 50 ng/mL, suggesting the immunomodulatory effect of vitamin D in reducing the risk of severe COVID-19. 25(OH)D3 is the form of the hormone most present in the circulation and is commonly measured to monitor a patient’s vitamin D status due to its long half-life and high concentration. The GERIA-COVID study of Annweiler et al. showed that an oral bolus of 80,000 IU cholecalciferol either in the week following the diagnosis of COVID-19 or during the previous month was associated in the frail elderly with less severe COVID-19 and a better survival rate at day 14 [142]. A further extension phase of this study was conducted to observe the three-month, all-cause mortality rate [143]. The same cohort of geriatric COVID-19 patients was treated with supplements of bolus cholecalciferol (50,000 IU per month, 80,000 IU or 100,000 IU or 200,000 IU every two–three months, or daily supplementation with 800 IU), and more supplemented participants survived at three months compared to those without vitamin D supplements. Despite the limited number of patients and various confounding factors in the analysis, compared with other studies, this one provided a long follow-up with analysis of longitudinal associations according to initial vitamin D treatment. The high-dose therapy was also tested by Ling et al., who studied the relationships between COVID-19 mortality and two potential predictors, 25(OH)D3 levels and cholecalciferol booster therapy, in almost 1000 acute COVID-19 hospital in-patients (444 in primary cohort, 541 in validation cohort) from three separate hospital centers [144]. Regardless of baseline serum 25(OH)D3 levels, treatment with cholecalciferol using high-dose booster therapy (approximately ≥ 280,000 IU) reduced the risk of mortality in acutely hospitalized patients of both the primary and validation cohorts. However, serum 25(OH)D3 levels were not associated with an increased risk of COVID-19 mortality, and vitamin-D-deficient status was associated with increased mortality only in the validation cohort. Ling et al. hypothesized that their study population might include good vitamin D responders with low serum 25(OH)D3 levels, as well as low responders with sufficient 25(OH)D3 serum levels; based on the concept of individual response to vitamin D, it is critical to identify these confounders, and it is difficult to establish a general cut-off dosage for all patients unless the cohorts studied are greatly enlarged. In the study by Giannini et al. [145], hospitalized, comorbid, fragile COVID-19 patients received 400,000 IU cholecalciferol, and the combined outcome of transfer to ICU and/or death was evaluated. The beneficial effect of cholecalciferol on outcome became gradually more pronounced with increasing comorbidity burden. In another study, patients with hypovitaminosis D were treated with 60,000 IUs of cholecalciferol for 8 or 10 days and were evaluated for inflammatory markers. The therapeutic improvement in vitamin D levels to 80–100 ng/mL significantly reduced the inflammatory markers associated with COVID-19 without any side effects [146]. A lower dosage of oral cholecalciferol (1000 IU/day) was administered to older COVID-19 patients, and it was demonstrated that the combined treatment with vitamin D/magnesium/vitamin B_12_ was associated with a significant reduction in the number of patients with clinical deterioration requiring oxygen support and intensive care support [147].

A possible reason for discrepancies reported in different studies could be the differences in dosage. In this regard, a randomized, clinical trial compared the effects of supplementation of 5000 IU (with 125 µg cholecalciferol) versus standard oral supplementation of 1000 IU (with 25 µg cholecalciferol) vitamin D3 per day among SARS-CoV-2-positive adults who were hospitalized for mild-to-moderate COVID-19 disease [148]. A short-term 5000 IU vitamin D3 supplementation reduced the recovery time of symptoms, particularly cough and ageusia and the loss of gustatory sensitivity, in patients with suboptimal vitamin D status compared to those treated with 1000 IU vitamin D3 and caused a significant increase in serum 25(OH)D3 levels [148]. The authors of the study hypothesized that vitamin D could ameliorate both these symptoms by the modulation of type 1 interferon receptors signaling and the consequent reduction of lung inflammation and cough and by the control of neurotrophins, proteins involved in the development of the gustatory system neurons [148]. In future clinical trials, high-dosage and short-term therapy should be tested in severe COVID-19 cases with a severe vitamin D deficiency. In another study, the patients were treated with different doses of cholecalciferol depending on their deficit (2000, 5000, and 10,000 IU) for at least 14 days. The treatment shortened hospital stay and decreased mortality in COVID-19 cases, even in the existence of comorbidities [149].

The administration of different metabolites of vitamin D demonstrates different efficacy. Oristrell et al. conducted an observational study on a large, population-based cohort to compare COVID-19 outcomes in patients supplemented in the period immediately preceding the diagnosis of COVID-19; they were either treated with formulations containing >250 μg of cholecalciferol or >250 μg of calcifediol per dose and were compared with untreated, matched controls. Patients who achieved 25(OH)D3 levels of ≥30 ng/mL through treatment with these vitamin D metabolites had a lower risk of SARS-CoV-2 infection, showed a lower risk of severe COVID-19, and a lower mortality compared to 25(OH)D3-deficient patients not receiving vitamin D supplements [150]. A good response to calcidiol was also reported by the retrospective study of Loucera et al., which found that patients who had been prescribed treatment preferentially with calcifediol (and less intensively with cholecalciferol) for other health objectives 15 days before hospitalization presented a better survival rate among COVID-19 patients [151].

The possible effect of calcifediol treatment on admission to ICU and related potential risk of death was analyzed in a pilot, randomized, clinical trial conducted by Castillo et al. None of the patients treated with a high dose of calcifediol (0.532 mg on entry and then 0.266 mg on day 3, 7, and weekly) died, and all were discharged without complications, while, among patients not treated with calcifediol, 50% required admission to the intensive care unit [152]. Similar results were obtained by other retrospective cohort studies employing calcifediol at the same dosage [153]. These studies, like those previously mentioned, offer an interesting therapeutic perspective on COVID-19 adverse outcomes. It was also suggested that the administration of calcifediol could also, in the future, be combined with dexamethasone or another corticosteroid, as they have potent, anti-inflammatory actions in the treatment of hospitalized COVID-19 patients [152]. The treatment with oral 25(OH)D3 revealed beneficial effects in improving immune function by increasing the lymphocyte percentage and decreasing the neutrophil-to-lymphocyte ratio in patients with COVID-19 that is associated with improved clinical outcomes [154].

Vitamin D supplementation was tested also in the form of calcitriol; in a pilot study, treatment with calcitriol 0.5 μg daily for 14 days resulted in the improvement of oxygenation, reduced need for ICU, and shorter length of stay among hospitalized patients with COVID-19 [155].

Although the results of these studies are promising, conflicting conclusions were reached in a few other trials. For instance, in the randomized, clinical trial conducted by Murai et al., a single high dose of 200,000 IU of cholecalciferol did not significantly reduce the length of hospitalization among patients with moderate-to-severe COVID-19 compared to the placebo. There was also no significant difference between the vitamin D3 group and placebo group for intra-hospital mortality or the need for mechanical ventilation [156]. However, one of the limitations of this study was the late treatment with cholecalciferol (a mean of ten days after symptom onset), which might have impaired the efficacy of the hormone. Further studies are needed to determine whether patients with COVID-19, especially those with mild or moderate disease, would benefit from preventive or early vitamin D supplementation. In another study by Güven et al., a small cohort of vitamin-D-deficient (25(OH)D3 < 12 ng/mL) patients, admitted to the intensive care unit with the diagnosis of COVID-19, was examined [157]. The treatment with a single intramuscular dose of 300,000 IU cholecalciferol in the early period of ICU admission did not result in improvements in the clinical course of patients. In fact, median hospital stay, the need for endotracheal intubation, and the in-hospital mortality rate were found to be similar between the vitamin D3 group and control group, suggesting that vitamin D3 may be ineffective in the critical stages of infection characterized by ARDS.

### 6.2. Concluding Remarks on Vitamin D Supplementation in COVID-19

The comparison between existing studies is made difficult by the heterogenous patient populations and the differences in treatment protocols. As was highlighted in Section 6.1, due to personal sensitivity to vitamin D activity, it is difficult to establish the proper dosage for an effective treatment of a whole cohort of patients; for this reason, the highest dose of hormone is probably the most appropriate choice, although the negative calcemic effects should be carefully evaluated, and non-calcemic analogs should be tested. It should be noted, however, that a large bolus of vitamin D may have minimal benefit or could even be counterproductive, whereas moderate daily doses in individuals at risk of deficiency would be more beneficial, as highlighted in most recent meta-analyses and reviews [158,159]. In addition to dosage and duration of treatment, it is important to assess the form of the metabolite administered. Cholecalciferol, native vitamin D3, is the most widely tested form due to its large availability and low cost, particularly in developing countries, as well as its relatively safe side-effect profile [144]. On the other hand, calcifediol appears to have advantages over cholecalciferol. Indeed, it does not require hepatic 25-hydroxylation, which can be hampered by liver overload; it has a more reliable intestinal absorption (close to 100%) and can rapidly restore serum concentration of 25(OH)D3 [150]. The advantage of calcidiol administration was reported in osteoporotic patients [160]. Furthermore, the best route of administration and early treatment must be considered, taking into account the clinical characteristics of the patients, baseline 25(OH)D3 levels, and outcome measurement [140,161]. Although some studies considered serum 25(OH)D3 as a negative acute-phase reactant [162], nonetheless, supplementation with the hormone was found to be generally helpful in COVID-19 outcomes. Further, large-scale population studies are needed to confirm the beneficial effects of vitamin D in reducing disease severity and in preventing infection.

## 7. The Biochemical Basis of the Antioxidant and Metabolic Activity of Vitamin D That Can Be Exploited in a Better-Integrated Therapeutic Approach

There are several comorbidities that increase the severity of COVID-19 symptoms such as obesity, diabetes, inherited glucose-6-phosphate dehydrogenase (G6PD) deficiency, and vitamin D deficiency. These pathological conditions have a common inflammatory and pro-oxidative background that increase the risk of exacerbated oxidative stress and impairment of specialized immune cell activities accompanied by a cytokine storm.

The antioxidant and anti-inflammatory activity of active vitamin D is exerted on different players of the pathways activated by SARS-CoV-2 infection. The possible molecular mechanisms by which vitamin D could alter host cell redox status and block viral entry were recently reviewed [163,164,165,166] in an effort to describe how vitamin D can prevent COVID-19 infection or reduce the severity of the disease. The reported mechanisms and further targets or the hormone can be summarized as:

1. Viral entry is hampered; the computational study by Song et al. demonstrated that the interaction of 1,25(OH)_2_D3 with the SARS-CoV-2 spike RBD (receptor-binding domain) causes a change in the dynamic movement of the binding surfaces between the SARS-CoV-2 RBD and the ACE2 that disrupts their binding [167]. In addition, it was seen that vitamin D and its hydroxyl derivatives bind the active site of TMPRSS2 (the transmembrane serin protease with the function of priming the spike protein) and inhibit SARS-CoV-2 RBD binding to ACE2 to prevent SARS-CoV-2 entry. Moreover, calcitriol downregulates the transcription of ACE2 codifying gene [168].
2. A recent proteomic study identified 332 human protein targets of the 27 viral proteins [169]. The investigation, carried out by genomics-guided tracing of SARS-CoV-2 targets in human cells, showed that vitamin D alters the expression of 84 genes out of 332 (25%) genes, encoding human proteins prey of 19 viral proteins. These observations suggest that vitamin D, in addition to the inhibition of ACE2 gene expression, may potentially interfere with the functions of 19 out of 27 (70%) SARS-CoV-2 proteins [168]. These analyses support the possibility that vitamin D and VDR are putative mitigation factors of the coronavirus infection.
3. Virus-triggered RAS imbalance is restored; active vitamin D can modulate the expression of the members of the RAS system in different pathological conditions where the system is altered [93,94,97,170,171]. In fact, in vitro analysis conducted on LPS-treated murine lung cells showed that high concentrations of calcitriol dramatically reduce the effects of LPS on ACE levels and decrease mRNA expression of ATR1 and AngII. Calcitriol also suppresses renin expression, resulting in the inhibition of the ACE/Ang II/AT1R cascade [97]. Moreover, in a study on obese mice, calcitriol administration attenuated acute lung injury complicated with sepsis by promoting the activity of the anti-inflammatory pathway in the RAS. This treatment induced higher ACE2 and MasR expression but lowered AT1R expression and decreased macrophage and neutrophil infiltration in the lung [170]. These studies support the conclusion that the imbalance of the RAS system correlates with the appearance of an uncontrolled inflammatory response [170] which can be reduced by active vitamin D.
4. The redox balance is maintained; vitamin D regulates the two main players of ROS production and redox defense: NF-kB and glutathione. In COVID-19, the increased levels of AngII generated by the inhibition of ACE2 activate NF-kB, which is responsible for the release of cytokines, enzymes of inflammation, and adhesion molecules. For these reasons AngII is considered an important inflammatory mediator. On the one hand, the NF-kB signaling pathway is partially blocked by active vitamin D [172,173,174]. On the other hand, ROS generated by the NF-kB signaling pathway are neutralized by the vitamin-D-dependent increased glutathione biosynthesis [33,175] and by the enhanced activity of various ROS-scavenging enzymes controlled by active vitamin D [163].
5. Mitochondrial oxidative stress is minimized; SARS-CoV-2 infection is associated with altered mitochondrial dynamics with consequent oxidative stress, which can be normalized by active vitamin D, preventing the pro-inflammatory state, cytokine production, and cell death [166]. Indeed, calcitriol through VDR plays a central role in protecting cells from excessive respiration and production of ROS that leads to cell damage [176].

In addition, it must be highlighted that some of the key metabolic switches triggered by SARS-CoV-2 can be counterbalanced by vitamin D action, further explaining its protective effects in COVID-19. In fact, several recent studies demonstrated the impact of the hormone on cellular metabolism, particularly on mitochondrial respiration. It was demonstrated that calcitriol reduces mitochondrial respiration [21,22,23] and uncoupling [177] and rewires cell metabolism toward the biosynthetic pathways, thus, exerting a protective effect against excessive respiratory activity and toxic ROS production [176]. To avoid oxidative stress, the respiratory chain must be tightly regulated and coupled to ATP synthesis. In fact, fluctuations in coupling the electron transport chain (ETC) activity to ATP production can lead to electron leak from the ETC, and unpaired electrons can react with oxygen to form ROS. Uncoupling proteins (UCPs) catalyze the net transfer of protons across the mitochondrial inner membrane, partially dissipating the protonmotive force as heat. On the one hand, UCPs have the function of decreasing protonic backpressure on the respiratory chain and lowering the production of ROS, the byproduct of respiration [178]; on the other hand, however, the uncoupled respiration reduces mitochondrial efficiency and leads to organelle damage [179]. Vitamin D activity can optimize cellular respiration by checking both ETC activity and UCP expression.

It is, therefore, reasonable to add at least two more mechanisms that could mediate the metabolic, antiviral activity of vitamin D:

6. The mitochondrial uncoupling triggered by viral infection can be reduced.

Viruses alter mitochondrial metabolism to maintain a suitable replication niche. Interestingly, viruses can induce different effects on the host metabolism, which specifically depend on the type of virus [180]. Several proteins of SARS-CoV-2 are predicted to interact with mitochondria, such as NSP4, NSP8, and ORF9c [169]. The distinct localization of viral RNA and proteins in mitochondria rewires the host cell’s mitochondrial function to viral advantage and must play essential roles in SARS-CoV-2 pathogenesis [181]. Mitochondrial uncoupling could be among the metabolic derangements triggered by viral infection. In fact, the interaction of spike protein with ACE2 stimulates the MAPK/NF-kB axis and results in the release of pro-inflammatory cytokines, such as TNF-α [182], which is an uncoupler agent [183,184]. For example, in oligodendrocyte progenitor cells, TNF-α alters mitochondrial calcium uptake, mitochondrial membrane potential, and respiratory complex I activity, as well as increases reactive oxygen species production [183]; moreover, TNF-α alters mitochondrial integrity by inducing an uncoupling effect in liver cells, as indicated by the reduction in membrane potential and by ATP depletion [184].

Calcitriol can restore the altered mitochondrial function by decreasing uncoupling through transcriptional repression of uncoupling proteins UCPs [177] and by limiting NF-kB signaling; both mechanisms contribute to prevent ROS production and oxidative stress, the most common causes of severe COVID-19.

7. The vesicle deacidification necessary for viral egress can be counteracted.

The viral protein ORF3a is involved in lysosomal deacidification [185]. Raising the pH of the lysosome allows the virus to use lysosomal organelles as vehicles to reach the plasma membrane and egress from the host cell [185]. Moreover, the pH-dependent, altered lysosomal function of infected cells can result in perturbation of antigen presentation and in disruption of endolysosomal toll-like receptor signaling, which requires acidification, leading to altered immune responses. Active vitamin D can facilitate acidification through the transcriptional induction of vacuolar ATPase (V-ATPase), which is reported in osteoclasts and macrophages [33,186]; the effect on vesicle acidification counteracts the activity of ORF3a and decreases virus exit.

Considering the few discordant results of treatment with vitamin D in COVID-19 and taking into account the multiplicity of pathways involved in the outcome of the disease, the efficacy of vitamin D could be increased by combination with other molecules that affect the mechanisms underlying severe COVID-19. For example, other antioxidant molecules or ROS scavengers could be associated with vitamin D to enhance its antioxidant effects. Alternatively, limiting the use of drugs that are eliminated by conjugation with glutathione, thus, consuming antioxidant defenses, such as paracetamol, could reinforce the antioxidant effects of vitamin D. Furthermore, the acidification of the vesicles could be increased by the combination of vitamin D and beta-glucans, as recently demonstrated in vitro [33], which could hamper the viral escape from infected cell.

A timely and proper supply of vitamin D, together with other molecules acting in synergy, could ameliorate its impact on severe symptoms or ease long-term COVID-19, which is possibly related to redox imbalance, inflammation, and energy metabolic defects [187].

## 8. Conclusions

Vitamin D is a known promoter of general health. Since numerous studies highlight its immunomodulatory and anti-inflammatory properties, and its use was proposed in COVID-19, we reviewed the evidence of correlation studies between vitamin D insufficiency and disease severity, and we discussed the limits of the supplementation trials, the results of which were collected during the pandemic. The expanded biochemical analysis of the mechanisms of action of vitamin D suggests that the hormone can act on several fronts in containing the viral infection and in decreasing the severe symptoms due to inflammation and oxidative stress. In the evolution of the pandemic, vitamin D, in association with other molecules that enhance its action, could be used to fortify the response to the virus and could help coexistence during endemic viral resurgences.

## Data Availability

Not applicable.

## Supplementary Materials

The following supporting information can be downloaded at: https://www.mdpi.com/article/10.3390/ijms23073621/s1.
