# Supplementary material for: The Role of Vitamin D in Supporting Health in the COVID-19 Era
Source: Int J Mol Sci. 2022 Mar 25;23(7):3621. doi: 10.3390/ijms23073621 (PMC8998275; doi:10.3390/ijms23073621)
Supplement: Supplementary file 1 [file ijms-23-03621-s001.zip › ijms-1619784-supplementary.pdf]

Table S1. Correlation between vitamin D levels and clinical course.

**Vitamin D deficiency and COVID-19 disease severity**				
**Author, year [Ref]**	**Study design**	**Sample features**	**Endpoints and results**	**Correlation found and limitations**
Vassiliou et al., 2021 [111]	Prospective observational study	30 ICU patients (80% male) with a mean age of 65 y, presenting symptoms 6 days prior to ICU admission; 20% vit. D insufficient and the others vit. D deficient.	**Clinical parameters**: demographic data, comorbidities (hypertension, hyperlipidemia, diabetes, ARDS), vital signs, vit. D levels, COVID-19-targeted compounds. **Results**: median vit. D levels of survivors higher than non-survivors; all dead patients belonged to low vit. D group.	Yes; **limitations:** Greek-only ethnicity, cohort of limited size. Potential confounders for low vit. D not considered. No vit. D measurements prior and successive to ICU admission.
Dissanayake et al., 2021 [117]	Systematic review/meta-analysis	72 observational studies, 4 randomized, controlled trials.	**Clinical parameters**: vit. D deficiency and insufficiency was defined based on thresholds, and cohorts were created accordingly. COVID-19 severity criteria chosen: hospitalization, hypoxia (invasive or non-invasive ventilation, presence of ARDS), death, and composite (all the previous criteria together). **Results**: 25(OH)D levels were lower in COVID-19 patients compared to controls; in severe COVID-19 patients compared to non-severe COVID-19; and in non-survivors compared to survivors.	Yes; **limitations:** high risk of bias and heterogeneity due to different timing in vit. D assessment, severe COVID-19 definition, and vit. D deficiency threshold.
Carpagnano et al., 2020 [87]	Retrospective observational study	42 patients with acute respiratory failure, mainly male; mean age: 74 y. 52% non-smokers, 43% former smokers, 2% currently smokers. 86% of patients presented at least one comorbidity. Most of the patients had severe or very	**Clinical parameters**: demographic data, medical history, comorbidities (hypertension, cardiovascular and kidney diseases, and diabetes), lab. tests (CRP, D-dimer, ferritin, IL-6). **Results:** IL-6 serum levels higher in severe vit.-D-deficient patients. Patients with severe vit. D deficiency < 10ng/mL after 10 days of hospitalization 50%	Yes; **limitations:** modest sample size, short follow-up of patients enrolled.

		severe hypossiemic respiratory failure. 81% had vit. D levels <30ng/mL.	mortality probability, ≥ 10 ng/mL had a 5% mortality risk; vit. D deficiency poor prognosis marker.	
Diaz-Curiel et al., 2021 [113]	Retrospective observational study	1549 patients; 835 male, 714 female; mean age: 70 y.	**Results**: lower vit. D levels associated with higher risk of ICU admission; vit. D deficiency and ICU admission risk independent of patients age and sex; deceased patients had lower vit. D levels compared to same age normal population.	Yes.
Vassiliou et al., 2020 [114]	Prospective observational study	30 ICU patients; 80% vit. D <19.9ng/mL, 20% vit. D 20–30ng/mL; 90% ARDS-positive; 80% male, mean age: 65 y.	**Clinical parameters**: comorbidities present: hypertension, hyperlipidemia, diabetes. **Results**: median vit. D levels higher for survivors.	Yes; **limitations:** 25(OH)D value assessed on ICU admission, no measurements prior or during the hospitalization period.
Subramanian et al., 2022 [115]	Retrospective observational study	472 patients, 360 alive and 112 deceased after 28 days of COVID-19. 56.8% male.	**Clinical parameters**: demographic data, BMI, and comorbidities. Measurement of serum 25(OH) D, vitamin D binding protein (DBP), and serum albumin. **Results:** increased mortality amongst patients with very low (<25nmol/l) or high (>100nmol/l) serum 25(OH)D compared with 25 (OH)D 50–74 nmol/L, selected as reference.	Yes; **limitations:** only included patients with available surplus sera, underpowered to evaluate any interaction between COVID-19 severity, vitamin D levels, and other known risk factors.
Sulli et al., 2021 [122]	Retrospective observational study	65 COVID-19 patients, 65 controls; half male, mean age: 76 y, all under oxygen therapy at entry (no ICU).	**Clinical parameters**: pulmonary deficit, respiratory parameters (PaO2, SO2, PaCO2, PaO2/FiO2), lab. parameters (25(OH)D, CRP, D-dimer, blood cell count, calcium, phosphorous, liver and renal function). **Results**: Vit. D levels lower in COVID-19 patients than in controls. Vit. D levels lower in deaths during hospitalization. Significant correlation between vit. D	Yes: **limitations:** small number of patients, only elderly patients needing hospitalization and oxygen therapy, not including mild disease or ICU patients. Different comorbidities present might interfere with the results.

			levels and PaO2, SO2, PaO2/FiO2; negative correlation between vit. D levels and O2%; no correlation between vit. D levels and PaCO2. Negative association between vit. D levels and severity of radiologic pulmonary involvement (CT-scan) and longer global disease duration. Vit. D negative correlation with D-dimer, CRP, and positive correlation with calcium levels.	
Munshi et al., 2020 [118]	Systematic review/meta-analysis	6 studies (2 Asian, 3 European, 1 American), 1378 COVID-19-positive patients; severe vs. non-severe outcomes, ICU vs. floor admission, living vs. dead.	**Clinical parameters**: vit. D, lab. tests, demographic data, comorbidities, complications. **Results:** patients with poor prognosis and outcome had lower vit. D levels. Vit. D deficiency plays an independent causative role in COVID-19 severity.	Yes; **limitations:** small number of eligible studies, bias, and heterogeneity between studies
Alguwaihes et al., 2021 [126]	Retrospective case-control study	222 individuals: 150 positive (97 males, 53 females) and 72 negative patients (38 males, 34 females).	**Clinical parameters**: demographical data, comorbidities, vit. D, serological tests (glycated hemoglobin, lipids, LDL, HDL, ALT, AST, LDH, renal blood urea nitrogen and creatinine, CRP and other inflammatory markers). **Results:** male sex is an independent risk factor for the pathology. Vit. D deficiency prevalent in all patients, no differences between groups. Severe vit. D deficiency is not associated with COVID-19 infection but is related to increased mortality risk.	Yes; **limitations:** lack of statistical significance in severe vit. D deficiency and mortality association (due to sample size). Selection bias since all hospitalized patients are affected by other, pre-existing conditions. Positive and negative cases not matched according to pre-existing conditions.
Bayramoglu et al., 2021 [124]	Retrospective cohort study	103 pediatric patients aged between 1 and 18 y; asymptomatic, mild, and moderate-to-severe group	**Clinical parameters**: vit. D levels, comorbidities (diabetes, asthma, tuberculosis, chronic renal failure), clinical data, lab. tests (CRP, procalcitonin, fibrinogen, D-dimer,	Yes; **limitations:** obesity effect on the clinical course and inflammation markers not evaluated.

		(hospitalized); mean age 12.2 y, 52.4% male. 41.7% vit. D deficient, 38.4% insufficient, and 18.4% sufficient.	ferritin, calcium, phosphorous, ALP), imaging (chest X-ray and computed tomography). **Results**: moderate-to-severe COVID-19 group had the lowest median vit. D levels and highest inflammation markers compared to other groups.	Causality not explained.
Petrelli et al., 2021 [119]	Systematic review/meta-analysis	43 studies, 612.601 patients	**Results**: vit. D deficiency is associated with severity and higher mortality rate.	Yes.
Saponaro et al., 2022 [120]	Retrospective observational study	93 COVID-19-related pneumonia patients (acute care units, patients not requiring intubation); 68.9% male patients, mean age 68 y; 21.5% patients having severe ARDS and 41.9% mild ARDS. 89% of patients had vit. D < 30ng/mL, 29% vit. D <10ng/mL. 16.1% patients died during the study period.	**Clinical parameters:** pneumonia confirmed by CT scans; cytokines analysis (IL-1β, IL-6, IL-10, TNF-α, MCPI-1/CCL2), CRP and D-dimer, gas exchange impairment used as index of disease severity; vit. D levels. **Results:** inverse correlation found between vit. D levels and IL-6, CRP, D-dimer, and IL-10. Inflammatory markers found significantly higher in patients with vit. D levels <20ng/mL. Vit. D insufficiency higher in patients with severe disease and ARDS. Vit. D levels lower in non-survivors compared to survivors.	Yes; **limitations:** no vit. D levels before hospitalization; it is not possible to define a causative role of vit. D depletion in severe COVID-19.
Anjum et al., 2020 [116]	Prospective observational study	140 severe COVID-19-diagnosed patients; 58.57% males, mean age: 42.46 y; BMI: 23.48 kg/m2. 58.57% of patients having vit. D < 25nmol/L. 15.71% of patients died during the study period.	**Results:** patients with vit. D levels < 25nmol/L had higher rate of mortality compared to non-deficient patients.	Yes.
Bakaloudi et al., 2021 [131]	Review	20 European countries were selected; more than 50% adult	**Results:** a correlation between severe vit. D deficiency and COVID-19 mortality rates was found.	Yes; **limitations:** data on vit. D deficiency not generated from national-level

		population vit. D deficient.		surveys. Annual average vit. D levels considered. Correlations between COVID-19 infection and age not performed. Vit. D levels assessed prior to COVID-19 pandemic.

Table S2. Correlation between vitamin D levels and infection risk.

**Vitamin D deficiency and Sars-CoV-2 infection susceptibility**				
**Author, year [Ref]**	**Study design**	**Sample size**	**Results**	**Correlation found and limitations**
Merzon et al., 2020 [112]	Population-based data study	14,000, 10% COVID-19-positive individuals.	Univariate analysis highlights association; multivariate analysis: age >50y, male gender, low–medium socioeconomic status positively associated with vit. D levels assessed only in presence of symptoms.	Yes (independent risk factor for both vit. D infection and hospitalization).
Dissanayake et al., 2021 [115]	Systematic review/meta-analysis	72 observational studies, 4 RCT.	Vit. D levels were lower in COVID-19 patients compared to controls.	Yes; **limitations:** high risk of bias and heterogeneity due to different timing in vit. D assessment, severe COVID-19 definition, and vit. D deficiency threshold.
Kaufman et al., 2020 [128]	Retrospective observational analysis	191,779 patients, median age 54 y,<br>68% female; SARS-CoV2 positivity rate 9.3%.	Lower SARS-CoV-2 positivity rates associated with higher circulating vit. D levels in the populations considered (black non-Hispanic, Hispanic patients). Younger patients showed higher positivity and lower mean vit. D levels compared to older ones (>60y). Male patients had higher positivity and lower mean vit. D levels compared to females.	Yes; **limitations:** SARS-CoV-2 testing based on presence, gravity of symptoms, and exposure to infected. Ethnicity estimates based on U.S. census proportions by zip code.

			Subjects having <20ng/mL of circulating vit. D showed a 54% higher positivity rate.	
Alguwaihes et al., 2021 [126]	Retrospective case-control study	222; 150 positive (97 males, 53 females) and 72 negative patients (38 males, 34 females).	Male sex is an independent risk factor for the pathology, while no differences in age and BMI were present. Vit. D deficiency prevalent in all patients, no differences between groups. Severe vit. D deficiency is not associated with COVID-19 infection.	No; **limitations:** selection bias since all hospitalized patients are affected by other, pre-existing conditions. Positive and negative cases not matched according to pre-existing conditions.
Petrelli et al., 2021 [119]	Systematic review/meta-analysis	43 studies, 612,601 patients.	In vit. D deficient subjects (<20ng/mL) the risk of COVID-19 infection is 50% higher compared to non-deficient subjects.	Yes.
Brandão et al., 2021 [103]	Retrospective observational study	13,930 individuals (Brazilian population); both males and females; age between 18 and 90 y; 2345 COVID-19 positive patients.	No differences between vit. D status and COVID-19 susceptibility. Clinical, environmental, socioeconomic, and cultural factors seem to have greater relevance than vit. D deficiency.	No; **limitations:** no comorbidities presence assessment.
Bakaloudi et al., 2021 [133]	Review	20 European countries were selected; more than 50% adult population vit. D deficient	A not significant positive correlation was found between COVID-19 infection and vit. D deficiency (<50nmol/L) and between COVID-19 infection and severe vit. D deficiency (<30nmol/L).	No; **limitations:** data on vit. D deficiency not generated from national-level surveys. Correlations between COVID-19 infection and age not performed. Vit. D levels assessed prior to COVID-19 pandemic. Vit. D concentrations considered were annual, averaged rated.

ICU: intensive care unit, ARDS: acute respiratory distress syndrome, RCT: randomized, controlled trial, CRP: C-reactive protein, IL-6: interleukin 6, PaO2: partial pressure of oxygen, SpO2: oxygen saturation, PaCO2: partial pressure of carbon dioxide, FiO2: fraction of inspired oxygen, CT-scan: computed tomography, LDL: low-density lipoprotein, HDL: high-density lipoprotein, ALT: alanine transaminase, AST: aspartate aminotransferase, LDH: lactate dehydrogenase, BMI: body mass index, IL-1β: interleukin 1 beta,

IL-6: interleukin 6, IL-10: interleukin 10, TNF-α: tumor necrosis factor-alpha, MCP-1: monocyte chemoattractant protein 1, CCL2: C-C motive chemokine ligand 2.

Table S3. Vitamin D supplementation tested in clinical trials.

Author, Year [Ref]	Sample Size	Administration protocol	Clinical Effects
Oristell et al., 2021 [151]	108,343	Formulations containing >250 μg of cholecalciferol or >250 μg of calcifediol per dose.	25OHD levels ≥30 ng/ml achieved with treatment were associated with lower risk of SARS-CoV-2 infection, lower risk of severe COVID-19, and lower COVID-19 mortality.
Rastogi et al., 2020 [142]	40	Daily 60000 IU of cholecalciferol (oral admin.) for 7 days. A weekly 60,000 IU provided to patients with 25(OH)D >50 ng/ml or continued with daily 60,000 IU for additional 7 days if 25(OH)D <50 ng/ml.	Asymptomatic or mildly symptomatic vitamin-D-supplemented patients achieved SARS-CoV-2 RNA negativity before day 21. A significant reduction of fibrinogen was observed in patients achieving 1,25(OH)2D3 >50 ng/ml.
Annweiler et al., 2020 [143]	66	Oral bolus of 80,000 IU vitamin D3 either in the week following the suspicion or diagnosis of COVID-19 or during the previous month.	This treatment in frail elderly was associated with less severe COVID-19 and better survival rate at day 14.
Annweiler et al., 2020 [144]	77	Oral bolus vitamin D3 (50,000 IU per month, 80,000 IU or 100,000 IU or 200,000 IU every 2–3 months, or daily supplementation with 800 IU).	Improvement in mortality in elderly patients at 3-month follow-up.
Sabico et al., 2021 [149]	69	5,000 IU (with 125 μg cholecalciferol) versus standard 1,000 IU (with 25 μg cholecalciferol) of vitamin D3 per day oral supplementation for 2 weeks.	5,000 IU, but not 1,000 IU, vitamin D3 supplementation reduced the recovery time for COVID-19 patients with mild-to-moderate symptoms.
Ling et al., 2020 [145]	444	Cholecalciferol high-dose booster therapy (approximately ≥ 280,000 IU) in a period of up to 7 weeks.	Reduction of the risk of mortality in acutely hospitalized patients admitted with COVID-19.
Entrenas Castillo et al., 2020 [153]	76	Oral calcifediol treatment: 0.532 mg on entry and then 0.266 mg on day 3, 7, and weekly until discharge or ICU admission.	Reduction of the need for ICU treatment of COVID-19 patients requiring hospitalization.

Alcala-Diaz et al., 2021 [154]	537	Calcifediol administration: 0.266 mg/capsule, 2 capsules on entry then one capsule on day 3, 7, 14, 21, and 28).	Treatment was significantly associated with reduced risk of 30-day mortality in patients hospitalized for COVID-19.
Tan et al., 2020 [148]	43	Single daily oral 1,000 IU dose of vitamin D3 (cholecalciferol), 150 mg of magnesium oxide, and 500 mg vitamin B_{12} (methylcobalamine) for 14 days.	Treated older patients had significantly lower need for oxygen therapy or intensive care support.
Giannini et al., 2021 [146]	91	400,000 IU bolus oral cholecalciferol: 200,000 IU administered in 2 consecutive days (the second and third day of the in-hospital stay).	Treatment can significantly improve the outcomes (ICU admission, mortality) in COVID-19 patients with comorbidities.
Loucera et al., 2021 [152]	16,401	Administration of cholecalciferol or calcifediol 15–30 days before the hospital admission.	Calcifediol, preferably, or cholecalciferol with a lower effect, were associated with a better survival rate among patients hospitalized because of COVID-19.
Elamir et al., 2022 [156]	50	Calcitriol 0.5 μg daily for 14 days.	There was an improvement in oxygenation and reduced need for ICU among hospitalized, adult patients with COVID-19.
Gönen et al., 2021 [150]	210	Cholecalciferol treatment: 2,000, 5,000, and 10,000 IU for at least 14 days.	Vitamin D treatment decreased the risk of hospitalization and mortality rate in COVID-19 cases even in the existence of comorbidities. The vitamin D action in COVID-19 might involve regulation of INOS1, IL1B, IFNg, cathelicidin-LL37, and ICAM1.
Lakkireddy et al., 2021 [147]	130	60,000 IU of vitamin D in the form of aqueol nano solution per day for 8 or 10 days.	There was a significant reduction of inflammatory markers (C-reactive protein, lactate dehydrogenase, IL-6, neutrophil/lymphocyte ratio) without any side effects in COVID-19 patients.
Maghbooli et al., 2021 [155]	106	Oral dose of calcifediol (25 μg) per day during hospital stay and 25(OH)D levels assessed in 2-month follow-up.	The therapy resulted in improved immune function by increasing blood lymphocyte percentage. No significant reduction in ICU duration and mortality in treated patients. Study underpowered for detecting significant differences in clinical outcome measures.

Murai et al., 2021 [157]	240	Single oral dose cholecalciferol of 200,000 IU.	Treatment did not reduce the length of hospitalization, intra-hospital mortality, or the need for mechanical ventilation compared with placebo. Limit of the study: vit. D treatment occurred a long time after symptom onset (mean of 10.3 days).
Güven et al., 2021 [158]	175	Single intramuscular dose of 300,000 IU vitamin D3.	No improvements of the clinical course of ICU admitted patients respective to control group. The markers of inflammation are not different between both groups.
